# A case of familial central precocious puberty caused by a novel mutation in the *makorin RING finger protein 3* gene

**DOI:** 10.1186/s12902-015-0056-8

**Published:** 2015-10-23

**Authors:** Anna Grandone, Grazia Cantelmi, Grazia Cirillo, Pierluigi Marzuillo, Caterina Luongo, Emanuele Miraglia del Giudice, Laura Perrone

**Affiliations:** Department of Woman, Child and General and Specialized Surgery, Seconda Università degli Studi di Napoli, Naples, Italy

## Abstract

**Background:**

Central precocious puberty (CPP) is often familial but its genetic cause is largely unknown. Very recently, the makorin RING finger protein 3 (*MKRN3*) gene, located on chromosome 15 in the Prader-Willi syndrome (PWS)-associated region (15q11-q13), has been found mutated in 5 families with familial precocious puberty. The *MKRN3* is a maternal imprinted gene and the phenotype is expressed only when the *MKRN3* mutations are localized on the allele inherited from the father. The function of this gene is not completely known and the phenotype caused by its defect is not yet fully elucidated. We report a new *MKRN3* mutation (Pro160Cysfs*14) causing familial CPP.

**Case presentation:**

The index case is a 7 years old girl showing Tanner stage 3 and pubic hair stage 1. Her bone age evaluated by TW2 method was 10.3 years. Her hormonal data confirmed the diagnosis of central precocious puberty. Familial medical history revealed precocious puberty in a cousin on paternal side. Paternal grandmother had menarche at the age of 9 years and 6 months and premature menopause when she was 36 years old. Genetic analysis revealed a new mutation (c477_485del; Pro160Cysfs*14) in the maternally imprinted *MKRN3*. Puberty onset was at 5 years in the other affected female family member. Precocious puberty was well controlled by pharmacological therapy.

**Conclusion:**

We expand the number of the *MKRN3* mutations associated with CPP and highlight the importance of an accurate family medical history to disclose the peculiar pattern of inheritance of this gene.

## Background

Pubertal timing is influenced by complex interplay among genetic, nutritional, environmental and socioeconomic factors [1, 2]. Population-based studies have provided compelling evidence supporting genetic effects on pubertal timing [3].

Central precocious puberty (CPP) is defined by the gonadotropin-dependent development of secondary sexual characteristics before the age of 8 years in girls and 9 years in boys. CPP is familial in about 25 % of cases, showing an autosomal dominant transmission with incomplete, sex dependent, penetrance [4]. Although recent genome-wide association studies have identified several loci associated with pubertal timing and age at menarche, the genetic causes of CPP are still largely unknown.

Mutations in the genes encoding kisspeptin I and its receptor represent the first identified genetic causes of CPP [5, 6]. Very recently, the makorin RING finger protein 3 (*MKRN3*) gene, located on chromosome 15 in the Prader-Willi syndrome (PWS)-associated region (15q11-q13), has been found mutated in 5 families with familial precocious puberty [7]. *MKRN3* is a maternal imprinted gene that is expressed only if transmitted from the father. Accordingly, *MKRN3* mutations are not associated with pathological phenotype if transmitted from the mother [8].

The function of this gene is not completely known. Probably, it is involved in the ubiquitination of the proteins and its action leads to the inhibition of factors promoting the pubertal pulsatile GnRH secretion [9]. Phenotype related to *MKRN3* mutations has not been completely elucidated given the paucity of the report published after the first description [10–13].

We report a new mutation (Pro160Cysfs*14) in the maternally imprinted *MKRN3* gene causing CPP in a girl and her cousin, and precocious menarche in her paternal grandmother.

## Case presentation

A girl of Italian origin was examined at age of 7 years for premature thelarche. The thelarche appeared when the patient was 6 years old.

When the girl came to our observation, she showed breast Tanner stage 3 and pubic hair stage 1. Her bone age evaluated by TW2 method was 10 years and 3 months. Anthropometric parameters are shown in Table 1. Fifth fingers clinodactily of hands and lumbar hyperlordosis represented additional clinical signs.

**Table 1:** Clinical and laboratory characteristics of the proband with the *MKRN3* mutation

Patient III.3		
At onset	Age ( Years)	6
At referral	Age (years)	7
	Weight (kg)	28.9 (25-50^th^)
	Height (cm )	136.6(>97^th^)
	Bone age (years)	10.3
	Tanner stage	B 3
	Pubarche stage	PH 1
Hormonal profile	Basal LH (IU/L)	4.1
	Basal FSH ( IU/L)	7.6
	Estradiol (pg/mL)	29.7
Pelvic ultrasound	Uterine transverse diameter (cm)	2.2
	Uterine length (cm)	4.0
	Left Ovary (ml)	2
	Right Ovary (ml)	2
Brain magnetic resonance imaging	Normal	

Diagnosis of CPP was performed on the basis of hormonal data and pelvic ultrasound (Table 1). Thyroid hormones and adrenal steroids were normal. Brain Magnetic Resonance (MRI) did not disclose any abnormality both at hypothalamic and pituitary level. Treatment with Gonadotrophin‐releasing hormone agonists analog (GnRHa) was started (3.75 mg of the long-acting GnRHa depot triptorelin once a month, intramuscular route).

**Fig. 1:**
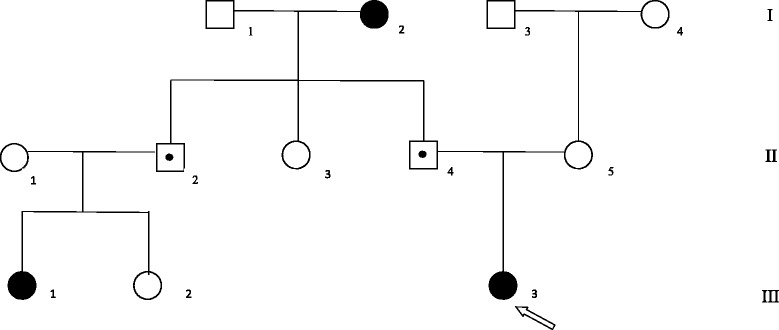
Pedigree of the family with the novel mutation of *MKRN3* gene. Squares indicate male family members. Circles indicate females. Black symbols indicate patients with CPP and, in case of the grandmother, with precocious menarche. Symbol with black points inside indicate the asymptomatic carriers. White symbols indicate non mutated patients. The arrow indicates the proband

Maternal menarche age (12 years) and father pubertal development were normal, but we identified a 35 years old female cousin belonging to the paternal side who showed hormonal confirmed central precocious puberty (LH 3.9 IU/L, FSH 4.2 IU/L, Estradiol 35 pg/mL) and premature menarche when she was 7 years old (Fig. 1). Her parents had both normal puberty onset. Considering this medical history, we suspected a genetic cause for the CPP, and, therefore, we focused on *MKRN3*. The extended family was investigated. Assent was obtained from the children and informed consent was obtained from the adults involved in the study. The ethical committee of the Second University of Naples approved the study.

Genomic DNA was extracted from peripheral blood leukocytes. The encoding region of the intronless *MKRN3* gene was amplified by overlapping primer pairs using standard PCR protocols, followed by direct sequencing of the purified PCR products.

We found a novel heterozygous mutation consisting in a deletion of 8 bp (c477_485del). It was a frameshift mutation and resulted in a premature stop codon leading to a truncated protein (Pro160Cysfs*14) with a predicted lack of both the C3HC4 RING motif and MKRN-specific Cys–His domain. Using the prediction models SIFT (Copyright 1993–2010, Fred Hutchinson Cancer Research Center, Seattle, WA, USA) and Provean (http://provean.jcvi.org), the P160Cfs*14 mutation has a deleterious effect consistent with the synthesis of a truncated and non-functional protein (score −13.5; threshold for deleterious effect: below −2.5). Both the proband and her cousin inherited the mutation from their fathers. In accordance with the expected pattern of inheritance, the fathers themselves reported to have normal puberty as they inherited the mutation from their mother (Fig. 1). Finally the grandmother reported precocious menarche (9 years and 6 months) and interestingly, premature menopause at 36 years. No clinical and biochemical data about the grandmother are available.

## Conclusion

Currently, 13 different loss-of-function mutations of *MKRN3* have been described in 30 patients with CPP from 17 multiplex families, including 7 frameshift defects, 4 missense mutations and 1 nonsense mutation [7, 10–13]. This kind of mutations, therefore, represent the most common cause of familial CPP.

We describe a family with CPP harboring a new mutation of the *MKRN3* gene. The frameshift mutation we found was located in the amino-terminal region of the protein, codified by a rich poly C site. In this region other three mutations have been found, suggesting that this area can be a potential hot spot.

A variable onset of precocious puberty has been shown in the mutated subjects of the same family. The paternal side female cousin (III.1 in Fig. 1) and the grandmother (I.2 in Fig. 1) presented menarche when they were 7 and 9 years old respectively, while the proband (patient III.3) presented thelarche onset at the age of 6 years. In addition, the proband showed mild dysmorphisms (i.e.; clinodactily and hyperlordosis). Among the other three reported patients carrying *MKRN3* loss of function mutations, one had clinodactily and hyperlordosis whereas the remaining two patients had esotropia [7, 11].

The mutated grandmother had also premature ovarian failure. This clinical feature has not been previously detected in patients with *MKRN3* mutations. Although the association with premature ovarian failure is purely speculative at the moment, it is interesting to note that in in PWS patients gonadal failure has been described as part of the clinical spectrum [14]. Of course, the link between the *MKRN3* mutation and premature ovarian failure has to be confirmed studying large cohorts of adult mutated patients.

In our case the familial nature of the CPP was evident only by the presence of a second degree and a third degree relative affected from the paternal side, as parents were asymptomatic and the girl had no siblings. Our finding confirms a previous suspicion that the familial nature of CPP can be frequently under-recognized if not carefully investigated [11]. Most children with CPP present for medical consultation accompanied by their mothers. Their paternal family history, therefore, may be often unknown, unavailable or under-investigated.

Due to maternal imprinting, only the paternal *MKRN3* allele is expressed. CPP, therefore, can manifest only if the mutated *MKRN3* allele comes from father. Nevertheless, fathers can be asymptomatic carriers if they have inherited the mutated allele form their mothers, as in our family. So we highlight the importance of an accurate family history that can unravel the familiar nature of CPP also in cases that seem sporadic. The determination of the precise onset of puberty in boys and in their fathers it is not simple since testicular enlargement is not as obvious as breast development and menarche in girls. So also when the father had precocious puberty, it could be not reported and only a very careful family medical history can reveal the presence of subjects with precocious puberty in second or third degree relatives.

Physicians should be aware of this kind of inheritance in CPP with the aim to promote *MKRN3* genetic analysis, providing an additional tool for the diagnosis and adequate treatment of children with CPP.

The identification and the long-term follow-up of new patients with *MKRN3* defects and CPP could be very interesting not only for the clarification of the phenotypes according with different kinds of mutations but also for establishing their risk of metabolic disorders and estrogen-dependent tumor development later in life.

### Consent

Written informed consent was obtained from the parents for publication of this Case report and any accompanying images. A copy of the written consent is available for review by the Editor of this journal.

## Abbreviations

MRI: Brain Magnetic Resonance
CPP: Central precocious puberty
GnRHa: Gonadotrophin‐releasing hormone agonists analog
*MKRN3*: Makorin RING finger protein
PWS: Prader-Willi syndrome
